# Evolution or Revolution in Colorectal Cancer Treatment: Present and Future of New Therapeutic Options. A Narrative Review

**DOI:** 10.32604/or.2025.067449

**Published:** 2026-01-19

**Authors:** Urszula Częścik, Martyna Gryglas, Arkadiusz Szterk, Sylwia Flis

**Affiliations:** 1 Centre of Postgraduate Medical Education, Doctoral School of Translational Medicine, Marymoncka 99, Warsaw, 01-813, Poland; 2 Center of Translational Medicine, Warsaw University of Life Science, Nowoursynowska 100, Warsaw, 02-797, Poland; 3 Division of Pharmacology and Toxicology, Department of Preclinical Sciences, Institute of Veterinary Medicine, Warsaw University of Life Sciences, Ciszewskiego 8, Warsaw, 02-786, Poland; 4 ASLAB Science P.S.A., Fort Służew 1/9, Warsaw, 02-787, Poland

**Keywords:** Colorectal cancer (CRC), treatment, chemotherapy, innovative therapeutic strategies

## Abstract

Colorectal cancer (CRC) is the third most common malignancy worldwide and the second leading cause of cancer-related deaths, accounting for approximately 10% of all cancer cases. By 2050, CRC incidence is expected to rise substantially, driven by population aging and greater exposure to risk factors in developing countries. Despite advances in medicine and pharmacy, the effectiveness of available treatments remains limited, underscoring the urgent need for innovative therapeutic strategies. This review summarizes and critically evaluates currently available CRC therapies and explores new emerging directions. Particular attention is given to the role of immunotherapy, targeted therapies, nanotechnology-based approaches, metal-based compounds, PROTAC technology, and personalized medicine, with emphasis on their efficacy, safety, accessibility, and mechanisms of drug resistance. In conclusion, surgery and chemotherapy remain the backbone of CRC treatment, but novel therapeutic approaches are reshaping the treatment landscape. Emerging strategies may offer improved patient tolerability and survival outcomes by reducing the occurrence of burdensome adverse effects. Persistent challenges such as drug toxicity, the emergence of resistance mechanisms, and inequalities in access to innovative therapies underscore the need for further translational research. Integrating personalized therapeutic approaches will also be crucial to achieving more effective, safer, and accessible treatment strategies for CRC.

## 1 Introduction

Colorectal cancer (CRC) develops in the colon or rectum, which form essential components of the digestive system. The process of carcinogenesis proceeds in several stages, starting with benign lesions, such as adenomatous polyps, which, as a result of the accumulation of genetic mutations involving the *APC*, *KRAS*, *TP53* genes, and the DNA mismatch repair (MMR) pathways, can transform into invasive lesions [1]. Colorectal cancer is the third most frequently diagnosed cancer in the world and one of the leading causes of cancer-related deaths. According to the Global Cancer Observatory (GLOBOCAN), in 2020, there were over 1.9 million new cases and about 935,000 deaths associated with CRC [2]. The incidence is highest in industrialized nations, especially Western Europe, Australia, and New Zealand, while the highest mortality rates are reported in Eastern Europe [2]. In the United States, CRC ranks fourth in incidence in both men and women. Annually, approximately 150,000 Americans are diagnosed with CRC, of whom more than 50,000 die [3,4]. The average age at diagnosis is 66 years, but there is a worrying increase in the disease being diagnosed in younger people. Recent projections indicate that by 2030, approximately 15% of all colorectal cancer cases will occur in individuals under the age of 50, with more than 27,000 such diagnoses expected annually in the United States [3,5]. Screening tests are of key importance in the prevention and early detection of CRC, which allow for the identification and removal of pre-cancerous lesions, significantly improving patient prognosis [6].

Metastases in CRC arise due to the migration of cancer cells from the primary tumor to distant organs, mainly via blood vessels or lymphatics. In the case of colon cancer, the liver is the primary metastatic site, which results from its direct vascularization via the portal vein. The second most frequently affected organ are the lungs, which the cancer cells can reach via the general circulation [7].

**Figure 1:**
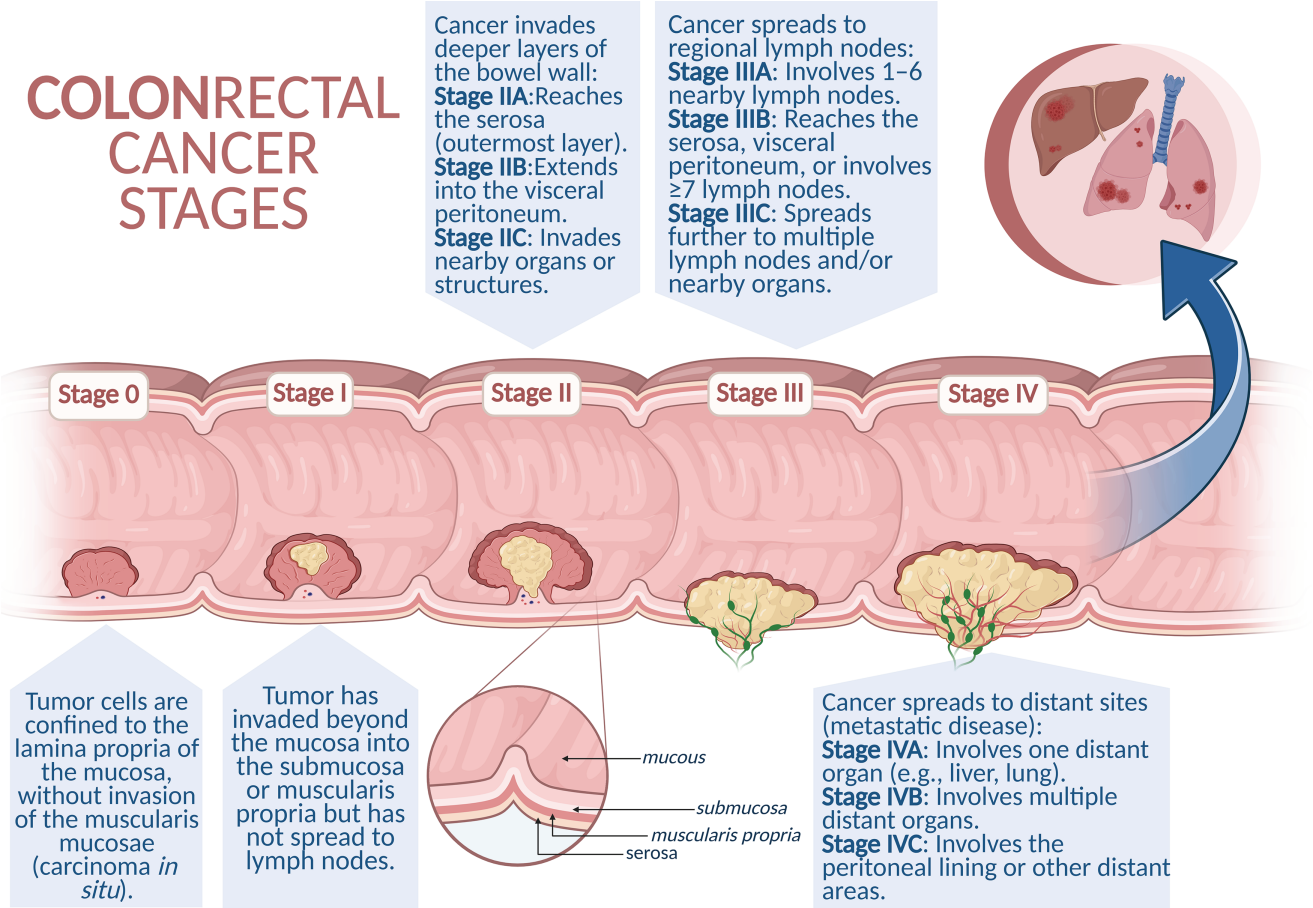
Schematic representation of colorectal cancer progression. Colorectal cancer develops through a stepwise process, beginning with non-invasive intraepithelial neoplasia (Stage 0), progressing to local invasion into deeper layers of the intestinal wall (Stages I–II), followed by regional lymph node metastasis (Stage III). In the most advanced stage (Stage IV), cancer cells spread to distant organs such as the liver, lungs, or peritoneum, representing metastatic disease. Created in BioRender. Cz, U. (2025) https://BioRender.com/skdi9m6 (accessed on 22 September 2025)

Colon cancer often remains asymptomatic for years. Hence, most patients are diagnosed in the advanced stage of the disease, when noticeable clinical symptoms manifest, such as persistent diarrhea, constipation, rectal bleeding or blood in the stool, abdominal discomfort (cramps, bloating, or pain in the lower abdomen), weight loss, chronic fatigue and general weakness [8]. The TNM (tumor-node-metastasis) classification developed by the American Joint Committee on Cancer (AJCC) is used to assess the clinical stage of the cancer. This system takes into account the size and depth of invasion of the primary tumor (T), the presence of metastases to regional lymph nodes (N), and the presence of distant metastases (M). Based on the TNM classification, the cancer is further classified into one of four clinical stages (I–IV), of which stages I–II are considered early and stages III–IV are advanced [9]. Correct classification and identification of the disease stage are crucial for selecting the optimal therapeutic strategy and determining the prognosis (Fig. 1).

## 2 Contemporary Approaches to the Treatment of Colorectal Cancer

**Figure 2:**
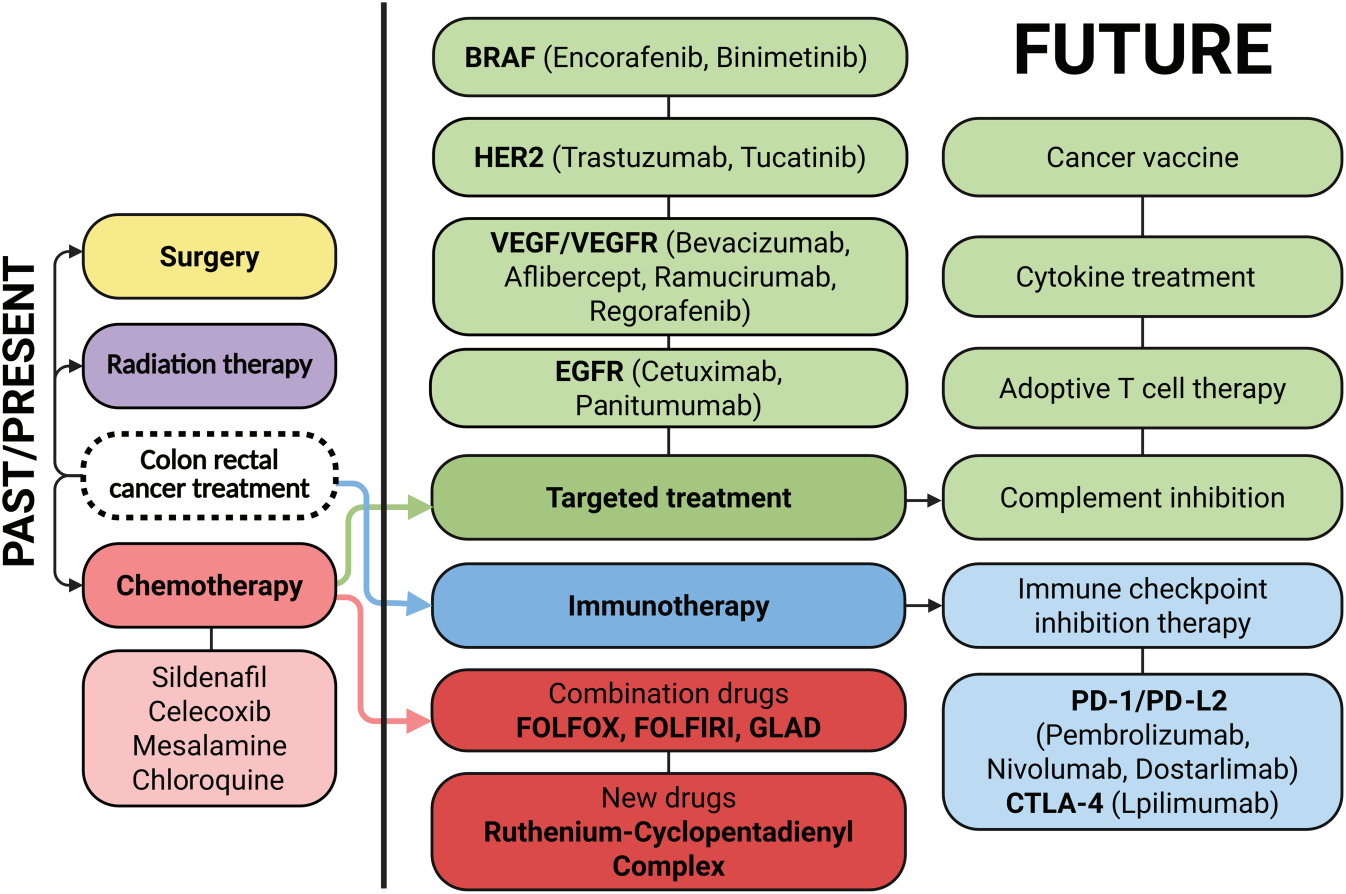
Therapeutic landscape and future directions in colorectal cancer treatment. Colorectal cancer management relies on a multidisciplinary approach, with surgical resection, radiotherapy, and systemic chemotherapy. Recent advances include targeted agents (e.g., VEGF, Vascular Endothelial Growth Factor; EGFR, Epidermal Growth Factor Receptor; BRAF, B-Raf Proto-Oncogene, Serine/Threonine Kinase; HER2 inhibitors, Human Epidermal Growth Factor Receptor 2 inhibitors) and immunotherapies (e.g., immune checkpoint inhibitors). Emerging approaches under investigation include cancer vaccines, adoptive T cell therapies, cytokine-based modulation, and complement system inhibition. Created in BioRender. Cz, U. (2025) https://BioRender.com/skdi9m6 (accessed on 22 September 2025)

Colorectal cancer continues to be among the most prevalent malignancies worldwide. The classic therapeutic approach is based on surgical resection complemented by chemotherapy and radiotherapy. In recent years, new strategies have also been developing dynamically, including targeted therapies, immunotherapy, and molecularly guided treatment modalities, all of which change the treatment paradigm for CRC patients [10,11] (Fig. 2).

Colorectal cancers are classified based on location, histopathological features, and molecular profile. Of particular importance is the anatomical and developmental division of the large intestine resulting from embryogenesis: the ascending colon and the right part of the transverse colon develop from the midgut, while the left part of the transverse colon, descending colon, sigmoid colon, and rectum develop from the hindgut. This corresponds to different vascularization (superior vs. inferior mesenteric artery), as well as biological and clinical differences, including response to treatment and prognosis [12].

Surgical resection remains the cornerstone of treatment of the neoplastic lesion together with regional lymph nodes. Techniques such as hemicolectomy, anterior resection of the rectum, or abdominoperineal resection of the rectum constitute the standard of care. In cases of rectal cancer, preoperative radiotherapy is also used, and in the case of colon cancer, adjuvant chemotherapy, particularly in patients at high risk of recurrence [13–15].

Chemotherapy remains the pillar of systemic treatment. The most commonly used drugs are fluoropyrimidines, 5-fluorouracil (5-FU) and its oral prodrug, capecitabine. The latter is activated in tumor tissue due to high levels of thymidine phosphorylase, which limits systemic toxicity and allows for effective oral treatment [16–18]. In the treatment of advanced CRC, FOLFOX (5-FU, leucovorin, oxaliplatin) and XELOX (capecitabine + oxaliplatin) are widely used regimens, which are characterized by comparable efficacy and tolerability [19–21].

One of the main challenges of therapy remains drug resistance. CRC cells show a high adaptive capacity, resistance may arise due to mechanisms such as efflux pump expression (e.g., P-gp), gene mutations, impaired prodrug activation, or diminished target protein expression [22,23]. Resistance is observed both *in vitro* and *in vivo*, and it concerns both classic cytostatics and modern targeted drugs. Therefore, intensive work is underway on new therapies, such as inhibitors of the epidermal growth factor receptor (EGFR) and vascular endothelial growth factor (VEGF), immunotherapy using monoclonal antibodies (e.g., cetuximab, bevacizumab) and molecularly targeted drugs for specific mutations, e.g., in the *BRAF* and *RAS* genes. Despite progress, chemotherapy remains an indispensable component of complex therapies and is often the first stage of systemic treatment, which can then be modified depending on the patient’s response.

## 3 Limitations of Colorectal Cancer Therapy

The main limitations of conventional therapy for colorectal cancer stem primarily from its substantial toxicity. Although cytotoxic agents are effective in eliminating rapidly proliferating tumor cells, their non-selective action results in damage to healthy tissues. Consequently, patients often experience adverse effects such as myelosuppression, peripheral neuropathy, nausea, vomiting, and diarrhea, significantly impairing their quality of life.

Another major challenge is the development of therapeutic resistance by cancer cells. As treatment progresses, cancer cells can activate adaptive mechanisms that reduce drug sensitivity, thereby promoting disease progression and narrowing the spectrum of effective therapeutic options [24]. A particularly concerning issue is the presence of colorectal cancer stem cells (CCSCs), which are inherently resistant to chemotherapy and play a critical role in tumor initiation, maintenance, and metastasis. Their capacity to evade conventional therapies contributes to tumor recurrence and prevents complete eradication of the disease [25].

These limitations significantly complicate the management of colorectal cancer and compromise long-term treatment efficacy. Consequently, current research efforts are focused on identifying novel, more effective treatment modalities with reduced toxicity profiles. Among these challenges, drug resistance has emerged as one of the most complex and multifactorial barriers to successful colorectal cancer treatment. Resistance to systemic therapies in CRC remains a major obstacle to achieving lasting responses and positive personalized treatment outcomes. Drug resistance mechanisms are typically classified as either primary (present before therapy initiation) or acquired (developing during treatment). These mechanisms encompass a wide range of molecular and cellular adaptations not only within tumor cells themselves but also in the tumor microenvironment (TME).

A common mechanism of intrinsic resistance involves the active efflux of chemotherapeutic agents by ATP-binding cassette (ABC) transporters, which actively pump out cytostatic drugs such as 5-fluorouracil, irinotecan, and oxaliplatin. A major player in this process is P-glycoprotein (P-gp; ABCB1, also known as MDR1), whose overexpression is frequently observed in CRC patients [26,27]. Unfortunately, clinical trials using ABC transporter inhibitors have thus far failed to yield satisfactory results, which has sustained interest in the search for novel, effective MDR inhibitors.

Another contributing factor to chemoresistance involves alterations in drug metabolism. CRC cells often display increased expression of metabolic enzymes such as glutathione S-transferases (GSTs), UDP-glucuronosyltransferases (UGTs), and cytochrome P450 (CYP) isoforms, all of which can enhance the inactivation or degradation of chemotherapeutic agents [28].

Acquired resistance is frequently associated with mutations in molecular targets and signaling pathway components. Activating mutations in *KRAS*, *NRAS*, and *BRAF* genes are well-established predictors of resistance to anti-EGFR monoclonal antibodies such as cetuximab and panitumumab [29,30]. In addition, activating mutations in the *BRAF* gene, particularly *BRAF*^*V600E*^, are linked to aggressive tumor biology and poor prognosis [31]. This mutation correlates with limited responses to anti-EGFR monotherapy and therefore necessitates the use of combination treatment regimens [32].

Activation of alternative pro-survival signaling cascades, such as RAS/RAF/MEK/ERK, PI3K/AKT/mTOR, and Wnt/β-catenin, enables cancer cells to bypass pharmacologic inhibition of primary oncogenic drivers. Dysregulation of these pathways contributes not only to anti-EGFR resistance but also to the maintenance of CCSCs, which exhibit inherent resistance to chemotherapy. This resistance is strongly associated with tumor repopulation and disease relapse [27,33]. CCSCs are particularly difficult to eradicate due to their slow proliferative rate, increased drug efflux activity, enhanced DNA repair capacity, and resistance to apoptosis. Therefore, targeting CCSC-specific signaling pathways remains a critical strategy in overcoming therapeutic resistance [34].

Epigenetic modifications, including DNA hypermethylation and histone modification, also regulate the expression of genes involved in apoptosis and drug metabolism. In addition, microRNAs (miRNAs) play a significant role in post-transcriptional gene regulation by promoting mRNA degradation or inhibiting translation. One of the most prominent miRNAs upregulated in CRC is miR-21, commonly referred to as an “oncomiR.” miR-21 has been shown to mediate resistance to 5-FU by downregulating components of the mismatch repair pathway. Similarly, miR-20a promotes resistance to 5-FU, oxaliplatin, and teniposide by downregulating BCL2/adenovirus E1B 19 kDa protein-interacting protein 2 (BNIP2) [35,36].

Lastly, though no less important, a critical contributor to the development and maintenance of therapy resistance in colorectal cancer is the tumor microenvironment, which provides a supportive niche for cancer cells. Cells within the TME stimulate the secretion of matrix-remodeling enzymes, cytokines, growth factors, and other anti-apoptotic and pro-metastatic mediators [37,38]. Moreover, the TME facilitates resistance development through multifaceted mechanisms, including immunosuppression, angiogenesis, and metabolic reprogramming. Hypoxia within the TME results in the stabilization and accumulation of hypoxia-inducible factor 1-alpha (HIF-1α), which upregulates the expression of genes encoding glucose transporters and glycolytic enzymes. The consequent increase in lactate production leads to acidification of the TME, thereby diminishing drug penetration and therapeutic efficacy. The activity of HIF-1α, together with nutrient deprivation (including glucose, glutamine, and tryptophan), significantly impairs the cytotoxic functions of CD8^+^ T cells and natural killer (NK) cells, while simultaneously promoting the activity of regulatory T cells (Tregs) and myeloid-derived suppressor cells (MDSCs). These changes collectively support tumor cell survival and exacerbate resistance to anticancer therapies [28,39,40].

The growing challenge of therapy resistance in CRC has spurred extensive research efforts to counteract its effects. Investigations into the molecular underpinnings of resistance have led to several promising therapeutic innovations. Notably, combination therapies targeting BRAF and EGFR alongside MEK or KRAS^G12C^ inhibitors have demonstrated encouraging results. Emerging epigenetic therapies employing DNMT and HDAC inhibitors are under active investigation, and immunotherapy has dramatically improved prognosis in patients with microsatellite instability-high (MSI-H) tumors. Additionally, nanoparticle-based drug delivery systems are being developed to enhance intratumoral drug accumulation and circumvent classical resistance mechanisms [41,42].

Ultimately, a deep understanding of the multifaceted resistance mechanisms in CRC, and the development of precision medicine approaches targeting these pathways remains essential for improving long-term treatment efficacy and tailoring therapy based on the molecular profile of individual tumors.

## 4 Innovative Therapeutic Strategies

### 4.1 Approved Targeted Therapies and Immunotherapies in CRC

Cancer immunotherapy and targeted therapies have revolutionized the treatment paradigm of CRC, offering more precise and individualized options compared to conventional chemotherapy (Table 1). Among the most notable advancements are immune checkpoint inhibitors (ICIs), monoclonal antibodies (mAbs) targeting specific signaling pathways, and kinase inhibitors aimed at key molecular drivers. These therapies have not only extended survival in selected subgroups of CRC patients but also opened new therapeutic avenues for those with previously limited treatment options.

**Table 1:** Key signaling pathways in colorectal cancer (CRC), corresponding drugs, and clinical relevance

Pathway	Biological role in CRC	Drugs/Inhibitors	Clinical notes	Reference
**Wnt/β-catenin**	Regulates cell growth, differentiation, and stemness; frequently mutated in CRC	Vitamin D, Lycopene, Genistein; Sulindac, Celecoxib, NSAIDs; LGK974, etc-159, OMP-18R5, Ipafricept; Niclosamide, Pimozide, Ethacrynic Acid	Investigated in the CORRECT trial (Regorafenib); a promising target in CSC-directed therapies	[43–45]
**PI3K/Akt/mTOR**	Promotes cell survival and proliferation; often hyperactivated in CRC	Alpelisib, Buparlisib, Pictilisib, Copanlisib, Pilaralisib	Investigated in clinical trials (e.g., NCT01719380, NCT01591421, NCT05862285) evaluating drug combinations	[43]
**Notch**	Maintains stem/progenitor cell populations; contributes to tumor progression and therapy resistance	DAPT; multiple Notch inhibitors (under investigation)	Targeted to enhance chemosensitivity; important in CSC maintenance	[46]
**Hedgehog/GLI**	Promotes cell proliferation and chemoresistance, particularly in KRAS/BRAF-mutated CRC	Vismodegib, GANT61, Arsenic Trioxide	Studied in combination therapy trials (e.g., NCT00636610)	[47,48]
**TGF-β/SMAD**	Exhibits dual roles: tumor suppressor in early stages and promoter of metastasis/stemness in later stages	Receptor kinase inhibitors, monoclonal antibodies (in trials)	Limited clinical success to date	[48]
**NF-κB**	Regulates inflammation and cell survival	–	Recognized therapeutic target, though specific inhibitors are not yet widely available	[49]
**JAK/STAT**	Modulates immune responses and cell proliferation	JAK inhibitors (under development)	Investigated in clinical trials (e.g., NCT02119676) in combination settings	[47,50]
**EGFR/VEGFR**	Promotes proliferation; reactivation frequently observed in resistance mechanisms	Cetuximab, Panitumumab	Standard-of-care in CRC treatment protocols	[43]
**CSC**	Associated with recurrence and metastasis; maintained via Wnt and Notch signaling	Targeted with Wnt/Notch inhibitors (e.g., LGK974, DAPT, Pimozide)	Focal point in ongoing clinical trials	[51,52]

Note: **CRC**, Colorectal Cancer; **Wnt**, Wingless/Integrated; **NSAIDs**, Non-Steroidal Anti-Inflammatory Drugs**; CSC**, Cancer Stem Cells; **PI3K**, Phosphoinositide 3-Kinase; **Akt**, Protein Kinase B; **mTOR**, mechanistic Target of Rapamycin**; DAPT**, N-[N-(3,5-difluorophenacetyl)-L-alanyl]-S-phenylglycine t-butyl ester; **GLI**, Glioma-Associated Oncogene Homolog; **KRAS**, Kirsten Rat Sarcoma Viral Oncogene Homolog; **BRAF**, B-Raf Proto-Oncogene, Serine/Threonine Kinase; **TGF-β**, Transforming Growth Factor Beta; **SMAD**, Mothers Against Decapentaplegic Homolog; **NF-κB**, Nuclear Factor kappa-light-chain-enhancer of Activated B Cells; **JAK**, Janus Kinase; **STAT**, Signal Transducer and Activator of Transcription; **EGFR**, Epidermal Growth Factor Receptor; **VEGFR**, Vascular Endothelial Growth Factor Receptor**; NCT**, National Clinical Trial Identifier.

Under physiological conditions, immune checkpoints maintain tolerance to self-antigens and prevent immune-mediated damage to healthy tissues during the response to pathogens. Cancer cells, however, have developed mechanisms to reduce or even completely suppress immune system activity by altering the expression of surface antigens. A reduced or absent expression of adhesion molecules necessary for T lymphocyte activation has been observed. The primary mechanism by which cancers evade immune detection appears to be the altered expression of immune checkpoint proteins. The use of antagonists targeting inhibitory immune checkpoint signals, as well as agonists of co-stimulatory receptors, can reinvigorate the antitumor potential of the immune system [53].

Immune checkpoint inhibitors (ICIs) represent a cornerstone of immunotherapy in CRC, particularly in tumors exhibiting high microsatellite instability (MSI-H) or deficient mismatch repair (dMMR). Agents such as nivolumab and pembrolizumab (anti-PD-1 antibodies), as well as ipilimumab (an anti-CTLA-4 antibody), have shown significant clinical efficacy in this subset of patients [54–56]. MSI-H/dMMR tumors, which account for approximately 15% of all CRCs and 5%–7% of metastatic cases, are characterized by a high tumor mutational burden (TMB), which enhances neoantigen presentation and facilitates T cell–mediated immune responses [55,56]. Although *BRAF* mutations, particularly V600E, are more commonly found in MSI-H tumors, and *KRAS* mutations in microsatellite-stable (MSS) tumors, it is the mismatch repair deficiency itself, and not *KRAS* or *BRAF* mutations, that primarily drives the elevated TMB observed in MSI-H CRC [56,57]. The phase III CheckMate-8HW trial confirmed the superiority of dual immunotherapy (nivolumab plus ipilimumab) over standard chemotherapy, demonstrating a 79% reduction in the risk of disease progression or death in treatment-naïve patients with MSI-H/dMMR metastatic CRC [56]. These results led to the European Commission’s approval of this combination as a first-line treatment in December 2024. Similarly, the NICHE-2 clinical trial, in which patients with dMMR nonmetastatic colorectal cancer received one dose of ipilimumab and two doses of nivolumab within 6 weeks before to surgery, demonstrated that 99% of patients achieved either a major (95%) or complete (67%) pathological response, indicating substantial tumor reduction or complete tumor clearance. Additionally, the study’s primary safety endpoint was met. Immune-related adverse events occurred in only 4% of patients, and surgery was delayed by ≥2 weeks in two cases. The evaluation of 3-year disease-free survival is ongoing (ClinicalTrials.gov identifier: NCT03026140) [58].

Nevertheless, ICIs are ineffective in microsatellite-stable (MSS) tumors, which represent the majority of CRC cases. This underscores the need for combinatorial approaches and novel biomarkers to predict immune responsiveness. Investigational strategies include combining ICIs with chemoradiotherapy, targeted therapies, oncolytic viruses, and cancer vaccines [59,60].

Targeted therapies directed against the EGFR and VEGF pathways have remained standard treatment options for metastatic CRC since their U.S. Food and Drug Administration (FDA) approval in 2004. Their efficacy is strongly influenced by the mutational status of the *KRAS*, *NRAS*, and *BRAF* genes. Anti-EGFR monoclonal antibodies such as cetuximab and panitumumab inhibit ligand-induced EGFR activation, thereby disrupting downstream MAPK and PI3K/AKT signaling pathways critical for cancer cell proliferation and survival. These agents are indicated exclusively for patients with wild-type *KRAS* tumors, particularly those with left-sided primary tumors, and are typically administered in combination with FOLFIRI or FOLFOX chemotherapy regimens [13,61,62]. Unfortunately, their use is associated with a spectrum of adverse events, including dermatologic toxicity (e.g., acneiform rash), hypersensitivity reactions, gastrointestinal disturbances (e.g., diarrhea), and impaired wound healing [63].

Importantly, resistance to anti-EGFR therapy often arises due to acquired mutations in *RAS* or *BRAF* genes. Current guidelines therefore emphasize the necessity of molecular profiling before initiating treatment. For instance, *BRAF*^*V600E*^-mutated tumors typically exhibit a poor response to anti-EGFR monotherapy but benefit from combination strategies involving BRAF inhibitors (e.g., encorafenib) and cetuximab, as demonstrated in the BEACON CRC trial [64].

Inhibition of the VEGF pathway by agents such as bevacizumab, aflibercept, and ramucirumab disrupts tumor angiogenesis, thereby limiting oxygen and nutrient supply, inhibiting tumor growth, and promoting vascular normalization. Bevacizumab, in particular, has shown benefit in both first- and second-line settings when combined with cytotoxic chemotherapy [65]. However, its use is associated with serious adverse events such as hypertension, thromboembolism, bleeding, gastrointestinal perforation, and impaired wound healing. Moreover, in the absence of reimbursement, the relatively high cost and variable efficacy dependent on VEGF expression remain limiting factors [14].

Aflibercept and ramucirumab offer additional options for patients with bevacizumab-refractory disease. Aflibercept, a recombinant fusion protein comprising the extracellular domains of VEGFR-1 and VEGFR-2, acts as a decoy receptor for VEGF-A, VEGF-B, and placental growth factor (PIGF), with a higher affinity for VEGF-A than bevacizumab. Although aflibercept monotherapy provides limited benefit, its combination with chemotherapy has demonstrated significant efficacy. The phase III VALOUR trial showed that adding aflibercept to FOLFIRI after failure with oxaliplatin or bevacizumab improved treatment outcomes in metastatic CRC [66]. Ramucirumab, a fully human IgG monoclonal antibody targeting VEGFR-2, was approved by the FDA for second-line treatment of metastatic CRC based on the RAISE trial [67]. Recently, the antiangiogenic agent fruquintinib has emerged as a promising option. Fruquintinib selectively inhibits VEGFR-1, -2, and -3, thereby blocking neovascularization. The phase III FRESCO-2 trial demonstrated that fruquintinib significantly improved both progression-free and overall survival in heavily pretreated metastatic CRC patients, regardless of prior therapy type [68].

The SUNLIGHT study further revealed that combining trifluridine/tipiracil with bevacizumab in patients with refractory metastatic CRC (mCRC) nearly doubled overall survival compared to trifluridine/tipiracil monotherapy (10.8 months vs. 7.5 months). This combination therapy was approved in both the European Union and the United States in 2023 (ClinicalTrials.gov identifier: NCT04737187) [69].

Another promising development is HER2-targeted therapy for HER2-amplified, RAS/BRAF wild-type CRC. The MOUNTAINEER study led to the approval of the tucatinib–trastuzumab combination for this subgroup, establishing a new biomarker-driven therapeutic strategy [70]. Likewise, KRAS^G12C^ inhibitors such as sotorasib, when used in combination with anti-EGFR agents, have shown encouraging clinical results and received regulatory approval in early 2025 for *KRAS*^*G12C*^-mutant CRC [71].

Finally, it is also worth mentioning regorafenib, a multi-kinase inhibitor introduced into the clinical setting of mCRC in recent years. It was originally developed as a RAF1 inhibitor, similarly to sorafenib; however, its spectrum of action is broader. Regorafenib induces wide-range drug sensitivity irrespective of the mutational status of major oncogenes, with its main therapeutic effects being anti-angiogenesis and remodeling of the tumor microenvironment. Despite promising anti-tumor effects in preclinical models, clinical benefits in mCRC patients have been modest. Randomized phase III trials, CORRECT (ClinicalTrials.gov identifier: NCT01103323) and CONCUR (ClinicalTrials.gov identifier: NCT01584830), showed that regorafenib significantly increased overall survival (OS) in heavily pretreated patients with mCRC, with a median survival benefit of 1.4 and 2.5 months over placebo, respectively. The CONSIGN phase IIIb study (ClinicalTrials.gov identifier: NCT01538680) further confirmed the safety profile of regorafenib in real-world settings [72].

After its approval by the FDA for the treatment of chemotherapy-refractory mCRC, several post-marketing observational studies highlighted a limited clinical benefit due to considerable toxicity. Consequently, ongoing research aims to optimize its clinical application. Recent studies focus on combining regorafenib with immune ICIs to enhance efficacy in MSS mCRC, a subtype typically resistant to immunotherapy. The REGONIVO study (ClinicalTrials.gov identifier: NCT04126733), an early-phase clinical trial that combined regorafenib with nivolumab and ipilimumab, has shown encouraging responses in MSS mCRC patients [73,74]. Another notable trial is a phase Ib/II study evaluating regorafenib plus toripalimab (anti-PD-1 antibody), which has reported manageable toxicity and preliminary signs of clinical activity [75]. These studies suggest that regorafenib may sensitize tumors to immunotherapy via modulation of the tumor microenvironment and gut microbiome. Further randomized trials are ongoing to validate these findings and establish the most effective and tolerable regorafenib-based combinations in advanced-line mCRC treatment.

In summary, approved targeted therapies and immunotherapies have significantly altered the treatment landscape of CRC. However, their efficacy is tightly dependent on precise molecular and biomarker profiling. Therefore, their rational implementation in clinical practice requires robust diagnostic infrastructure and continued research efforts to broaden their applicability beyond currently defined genomic subtypes.

### 4.2 Future Targets and Investigational Immunotherapies

As the limitations of current CRC treatments persist, extensive research continues to focus on identifying novel molecular targets and immune-related pathways that could provide therapeutic benefits across a broader patient population. Several experimental immunotherapeutic and targeted strategies are currently undergoing preclinical evaluation or early-phase clinical trials, aiming to overcome resistance mechanisms and address the molecular heterogeneity of CRC.

A major area of investigation involves the modulation of tumor-associated macrophages (TAMs) and myeloid-derived suppressor cells, both of which contribute to immune evasion within the tumor microenvironment. Agents targeting the colony-stimulating factor 1 receptor (CSF1R), such as pexidartinib and cabiralizumab, are being explored to deplete TAMs and reprogram the TME toward a more immune-permissive phenotype [39,76]. Early-phase clinical trials in solid tumors have shown promising results, although specific efficacy in CRC remains under investigation [77,78].

Another promising possibility involves bispecific antibodies, which are designed to simultaneously bind two different antigens. For instance, CEA-TCB (cibisatamab), a T-cell engager targeting carcinoembryonic antigen (CEA) on tumor cells and CD3 on T cells, has demonstrated tumor-specific immune activation both *in vitro* and in early-phase clinical trials for CRC [79,80]. These agents aim to enhance cytotoxic immune responses by directing T cells specifically to tumor cells, thereby minimizing off-target effects.

Emerging therapies are also targeting the Wnt/β-catenin and Notch signaling pathways, which play critical roles in maintaining stemness, promoting therapy resistance, and contributing to immune exclusion in CRC. Small molecules such as ETC-159 and the γ-secretase inhibitor DAPT are in development as Wnt/Notch pathway inhibitors. These pathways are particularly relevant to cancer stem cell biology, which is associated with tumor recurrence and poor response to conventional chemotherapy (ClinicalTrials.gov identifier NCT02521844) [46,81–83].

Another noteworthy target is the stimulator of interferon genes (STING) signaling pathway. STING agonists aim to activate innate immunity by inducing the production of type I interferons (IFN-I), enhancing antigen presentation, and supporting adaptive immune responses. Recent studies have demonstrated that activation of the cGAS–STING pathway in CRC stimulates the expression and secretion of IFN-I and pro-inflammatory cytokines, which in turn enhances CD8^+^ T-cell activation, promotes antitumor immune responses, and inhibits CRC progression. Consequently, targeting the cGAS–STING pathway and developing modulatory agents hold significant promise for improving therapeutic outcomes in CRC. Although clinical data in CRC are still limited, STING-based therapies are being explored, including in combination with immune checkpoint inhibitors, particularly to potentiate immune responses in MSS tumors [84,85].

The gut microbiota has also emerged as a key factor influencing cancer immunotherapy outcomes. Preclinical studies indicate that the composition of the gut microbiota significantly affects both ICI response and chemotherapy efficacy. In murine models, much of the focus has been on how gut microbiota modulate adaptive immunity during ICI treatment. One proposed mechanism involves enhanced CD8^+^ T-cell–mediated antitumor responses facilitated by specific microbial populations. Ongoing studies involving fecal microbiota transplantation (FMT), defined bacterial consortia, and engineered probiotics are evaluating their potential to modulate systemic immunity and improve therapeutic outcomes in CRC [86–88].

Despite its success in several malignancies, immunotherapy alone has limited efficacy in pMMR/MSS/MSI-L CRC. ICIs have shown minimal clinical benefit in this subset of patients, emphasizing the need for combinatorial strategies. Oncolytic viruses (OVs) represent a novel therapeutic platform with dual functionality: direct lysis of tumor cells and immunomodulation of the TME. Importantly, OVs can convert “cold” tumors, those lacking immune cell infiltration, into “hot” tumors, which are more responsive to ICIs. This is achieved by promoting tumor antigen release, stimulating pro-inflammatory signaling, and recruiting immune effector cells. Current preclinical and clinical studies investigating OV–ICI combinations have yielded encouraging results, particularly with genetically engineered OVs expressing immunostimulatory molecules [89,90].

Summarizing, these investigational approaches highlight a growing shift toward the integration of systems biology and immuno-oncology to develop multimodal CRC therapies. While many of these strategies remain in early stages of development, they hold considerable promise for overcoming the limitations of current treatments, especially in patients with MSS and treatment-refractory disease. Ongoing clinical trials and translational studies will ultimately determine which of these strategies may evolve into future standards of care.

### 4.3 Heavy Metal-Based Compounds: Mechanistic Insights and Limitations

While the exploration of metal-based compounds in cancer therapy has a long-standing history, including the well-established use of platinum-based drugs such as cisplatin, carboplatin, or oxaliplatin in CRC, recent research has shifted attention toward alternative metals like gold (Au), ruthenium (Ru), and copper (Cu). These efforts are driven by the need to overcome platinum-associated toxicities and resistance mechanisms. However, despite promising *in vitro* and *in vivo* data, no metal-based compound beyond platinum (Pt) has yet demonstrated sufficient efficacy or safety to be approved for clinical use in CRC patients.

Oxaliplatin, a third-generation platinum compound, remains the only metal-based cytotoxic agent approved for CRC. It is widely used in first-line regimens such as FOLFOX and XELOX. Although oxaliplatin is effective, it is associated with dose-limiting peripheral neuropathy, hematologic toxicity, and gastrointestinal side effects [91,92]. Moreover, resistance to platinum agents remains a significant hurdle, prompting the search for alternative metal-based therapeutics.

Gold (III) compounds have garnered considerable interest as promising alternatives to Pt-based anticancer drugs due to their structural similarity [93]. However, the mechanism of action of gold complexes is different. Studies indicate the primary target for gold complexes is not DNA, but certain proteins, such as thioredoxin reductase (TrxR). This is important, because in colon cancer, the thioredoxin system, consisting of thioredoxin-1 (Trx1) and thioredoxin reductase, is crucial for redox homeostasis, cellular proliferation, and the suppression of apoptosis. Numerous studies have shown overexpression of *TRX1* and *TRXR* genes in colon cancer tissues. Their elevated levels correlate with clinical advancement, metastasis, and poorer prognosis [22,23,94,95]. Gold compounds, especially auranofin and other Au(I)/Au(III) complexes, have shown cytotoxic effects in CRC cell lines primarily through the inhibition of thioredoxin reductase, leading to oxidative stress and apoptotic cell death [96,97]. Preclinical studies indicate that these compounds may sensitize tumor cells to chemotherapeutics and radiotherapy [98,99]. Nevertheless, their clinical translation is hindered by poor solubility, off-target toxicity, low bioavailability, and lack of human efficacy data.

Similarly, ruthenium-based complexes have emerged as potential antitumor agents due to their favorable pharmacokinetics and redox activity. Ruthenium-based complexes, particularly with a cyclopentadienyl ligand, present several advantages, such as higher specificity and lower cytotoxicity. Studies have shown that Ru complexes exhibit synergistic effects in combination with established anticancer agents and drugs. Moreover, some Ru complexes exhibit photoactivated cytotoxicity, DNA intercalation, or inhibition of topoisomerase II, they are widely used as phototherapeutic agents, biomolecular probes, and bioimaging reagents. Luminescent Ru complexes can differentiate DNA structures and have the potential to be used as molecular light switches for DNA [100,101]. Although these properties are attractive for therapeutic design, to date no ruthenium compound has advanced beyond early-phase trials in CRC [101,102].

Despite promising preclinical results, the enthusiasm surrounding metal-based compounds must be tempered by the reality of translational limitations. None of the non-platinum metal complexes have reached phase III trials or demonstrated clinical benefit in CRC populations. Toxicity profiles, formulation challenges, and unpredictable biodistribution further complicate their development. Therefore, while the mechanistic rationale for exploring gold, ruthenium, or copper complexes is scientifically valid, these compounds remain investigational and should be interpreted as adjuncts to, rather than replacements for, established therapies. Their future clinical utility depends on overcoming substantial pharmacological and toxicological barriers. Further studies, especially those integrating nanotechnology or targeted delivery systems, may help unlock their potential in a clinically meaningful way.

### 4.4 Nanotechnology-Enabled Drug Delivery in Colorectal Cancer

Nanotechnology has emerged as a promising platform for advancing cancer therapeutics, including CRC, by enabling precise drug delivery, improved pharmacokinetics, and reduced systemic toxicity. Nanocarrier-based systems are being actively developed to address the limitations of conventional chemotherapeutics and enhance the efficacy of targeted therapies and immunotherapies.

Nanoparticles (NPs), including liposomes, polymeric micelles, dendrimers, metal-organic frameworks, and albumin-bound drug carriers, offer several advantages. Their small size (typically 10–100 nm), tunable surface characteristics, and enhanced permeability and retention (EPR) effect enable preferential accumulation within tumor tissues. The EPR effect is especially relevant in CRC, where the abnormal vasculature and poor lymphatic drainage of tumors support selective NP retention [103,104].

Several nanoformulations have entered preclinical (*in vitro* and *in vivo*) and clinical evaluation. Recent preclinical studies were conducted by Huang and colleagues, who encapsulated high concentrations of irinotecan (IRI) in liposomes (Lipo-IRI) using the thin-film hydration method. *In vivo* studies in a tumor-bearing mouse model included pharmacokinetic assessments, biodistribution, safety, and therapeutic efficacy of Lipo-IRI. Pharmacokinetic profiling indicated that Lipo-IRI improved drug kinetics and prolonged circulation time compared to free IRI. While free IRI was rapidly eliminated via the mononuclear phagocyte system, Lipo-IRI exhibited a longer half-life and increased area under the curve (AUC). Moreover, the clearance of Lipo-IRI was reduced by 4.8-fold compared to free IRI, suggesting an extended residence time in the bloodstream. Notably, a 5 mg/kg dose of Lipo-IRI demonstrated antitumor activity without inducing colonic inflammation. Additionally, Lipo-IRI showed superior tumor accumulation compared to free IRI [105].

In another study, a novel oral drug delivery system was developed using solid lipid nanoparticles (SLNs) co-loaded with irinotecan and the isoflavonoid daidzein (DZN). The nanoparticle surface was functionalized with targeting ligands, including hyaluronic acid (HA), bovine serum albumin (BSA), and chitosan, to enable active targeting of receptors expressed on the colonic epithelium. Orally administered HA-BSA-coated SLNs, further protected by a chitosan layer, effectively targeted colon cancer cells via CD44 and SPARC receptors. This formulation ensured high drug entrapment efficiency, biocompatibility, and protection from gastrointestinal degradation. *In vivo* studies demonstrated reduced tumor markers and restored normal colon histology. This natural–synthetic nanoparticle system achieved approximately 90% targeted delivery and holds promise for safe and effective colorectal cancer therapy [106].

One clinical trial involving liposomes has been completed. Since 2005, a phase I study has investigated CPX-1, a novel liposome-encapsulated formulation of irinotecan and floxuridine. The study aimed to determine the maximum tolerated dose and pharmacokinetic profile of CPX-1. Initial findings indicated that the recommended dose for future trials should not exceed 210 units/m^2^, as neutropenia and diarrhea were identified as dose-limiting toxicities. Disease control, defined as complete response, partial response, or stable disease, was observed in 11 of 15 patients (73.3%) with colorectal cancer. A multicenter trial of CPX-1 liposomal injection in patients with advanced colorectal cancer is currently ongoing to evaluate its efficacy and safety (ClinicalTrials.gov identifier: NCT00361842).

In addition, the CRACK II phase study evaluated the efficacy and safety of cetuximab, camrelizumab, and liposomal irinotecan (HR070803) in patients with RAS wild-type metastatic colorectal cancer previously treated with anti-EGFR therapy. Patients received cetuximab (500 mg/m^2^), camrelizumab (200 mg), and liposomal irinotecan (60 mg/m^2^) every two weeks. Among 16 evaluable patients, the objective response rate (ORR) was 25%, and the disease control rate (DCR) was 75%. Median progression-free survival (PFS) and overall survival (OS) were 6.9 and 15.1 months, respectively. Grade 3 treatment-related adverse events (TRAEs) occurred in 15.8% of patients, with no grade ≥4 events reported. These findings suggest that this combination may represent a promising late-line therapeutic option with manageable toxicity in RAS wild-type mCRC [107].

Other NP-based systems, such as polymer-drug conjugates (PDC), are under investigation for their potential to prolong circulation time and control release kinetics. One widely investigated copolymer is polyethylene glycol (PEG), due to its hydrophilic properties and biocompatibility. PEG is approved for human use by the US Food and Drug Administration because of its unique properties, such as low antigenicity, lack of immunogenicity, high solubility in water and in organic solvents and minimal toxicity [108]. Presently, there are two ongoing clinical trials using PEG as a carrier in the treatment of CRC. The first one, a phase 2 study, is conducted to evaluate the efficacy and safety of NKTR-102 (PEG-IRI) vs. IRI in mCRC patients (ClinicalTrials.gov identifier NCT00856375), while the second study (07-PIR-02), also a phase 2 study, is designed to evaluate the safety and efficacy of NKTR-102 in combination with cetuximab for the treatment of patients with CRC (ClinicalTrials.gov identifier NCT00598975), but there are no final results yet.

A new approach in nanotechnology is the usage of albumin nanoparticles with docetaxel (Apt-NPs-DTX), which demonstrate targeted drug delivery to tumor cells, good biocompatibility, and prolonged drug release, resulting in enhanced therapeutic outcomes [109].

The primary concept behind a targeted drug delivery system (TDDS) is to selectively transport cytotoxic agents to tumor tissues while minimizing exposure to normal tissues. This approach enhances therapeutic efficacy and reduces adverse effects. To increase the likelihood of clinical translation, a TDDS designed for colorectal cancer treatment should demonstrate excellent biocompatibility, with nanoparticles (NPs) preferably composed of FDA-approved excipients.

Albumin, a water-soluble protein approved for human use by the FDA, is a promising excipient in this context. Albumin-based NPs exhibit outstanding biocompatibility, characterized by non-toxicity, low immunogenicity, and favorable biodegradability. Moreover, these NPs often enable sustained release of anticancer drugs, thereby improving pharmacokinetic profiles and drug utilization. Notably, an albumin-based formulation of paclitaxel (Abraxane^®^) has received FDA approval for the treatment of metastatic breast cancer, non-small cell lung cancer, and pancreatic adenocarcinoma. This supports the rationale that such a delivery strategy could also be applicable to CRC. Yu and colleagues were the first to develop an albumin-based TDDS (Apt-NPs-DTX) for targeted delivery of docetaxel (DTX) to colon cancer cells. This system utilizes a DNA aptamer (AS1411) as the tumor-targeting ligand, which specifically binds to nucleolin, a protein overexpressed on the surface of colon cancer cells. It is also noteworthy that DTX is an FDA-approved, broad-spectrum anticancer agent. In both *in vitro* and *in vivo* studies, the Apt-NPs-DTX formulation demonstrated superior efficacy against colon cancer cells compared to non-targeted delivery systems [109].

Based on the success achieved with the delivery of single chemical agents, the co-delivery of two distinct chemotherapeutic drugs has been developed and clinically implemented for the treatment of various types of cancer. A variety of nanoparticles has been investigated to design novel co-delivery systems, which can broadly be categorized into inorganic-based and organic-based NPs. Inorganic-based NPs primarily include mesoporous silica nanoparticles, iron oxide nanoparticles, metallic nanoparticles (e.g., copper, gold, or silver), and quantum dots. In contrast, organic-based NPs comprise polymeric micelles, polymeric nanoparticles, liposomes, dendrimers, and related structures. Furthermore, nanotechnology platforms enable the co-delivery of synergistic drug combinations or the incorporation of therapeutic biomolecules such as siRNA, miRNA, CRISPR/Cas9 components, and immunomodulatory agents. These capabilities support the concurrent modulation of multiple oncogenic pathways, immune checkpoints, and resistance mechanisms. In preclinical CRC models, enhanced therapeutic efficacy and immune reactivation have been demonstrated using nanocarriers loaded with antibody ligands, prodrugs, or peptide ligands [110].

Despite these promising developments, the clinical translation of nanomedicine in CRC remains challenging. Key obstacles include manufacturing scalability, tumor heterogeneity affecting nanoparticle uptake, and the lack of robust predictive biomarkers for selecting patients most likely to benefit. Additionally, regulatory hurdles and the complex pharmacokinetic behavior of nanoparticle formulations further complicate clinical development.

In conclusion, nanotechnology represents a versatile and potent strategy for optimizing CRC treatment. Although several nanotherapeutic agents have entered clinical practice, continued advances in material science, tumor-specific targeting, and rational combination therapies are critical to fully harnessing their potential in the era of personalized oncology.

### 4.5 Proteolysis-Targeting Chimera (PROTAC) Technology: A Breakthrough in Selective Protein Degradation

Currently, most approved small-molecule drugs act via occupancy-driven pharmacology, a mechanism of action (MOA) in which therapeutic efficacy depends on the duration and extent of target engagement, typically resulting in transient inhibition of protein function. Unfortunately, MOA cannot be applied to all biological targets, especially those that lack enzymatic activity, such as scaffolding proteins or proteins that function via protein–protein interaction. In response, translational efforts have increasingly focused on the development of next-generation therapeutics with alternative mechanisms of action, designed to engage non-traditional molecular targets and overcome established resistance pathways. This paradigm shift has broadened the therapeutic landscape to include modalities such as nucleic acid-based agents, engineered peptides, recombinant proteins, and monoclonal antibodies. In this context, proteolysis-targeting chimeras (PROTACs) represent a novel class of small-molecule therapeutics that induce selective degradation of disease-associated proteins via the ubiquitin–proteasome system. This bifunctional approach, which links a ligand for a target protein with an E3 ubiquitin ligase recruiter, enables targeted protein knockdown rather than simple inhibition, offering an innovative strategy for undruggable targets in oncology, including colorectal cancer. Unlike conventional inhibitors that require sustained binding to inhibit protein function, PROTACs act catalytically, redirecting E3 ligases to specific substrates to induce polyubiquitination and subsequent proteasomal degradation. This allows lower dosing, reduced off-target effects, and the potential to overcome resistance mechanisms driven by target overexpression or mutation [111,112]. In the context of CRC, emerging studies suggest that PROTACs might effectively degrade key oncogenic drivers such as mutant *KRAS*^*G12C*^ [113], BCL-XL [114], BET family proteins [115], and CDK proteins [116].

Importantly, the PROTAC approach offers a novel strategy for targeting previously intractable proteins, such as scaffolding molecules and transcription factors, by exploiting transient binding interactions. In colorectal cancer models, PROTAC molecules are being investigated for the targeted degradation of components of the Wnt/β-catenin signaling pathway, thereby overcoming resistance to conventional Wnt inhibitors. Previous studies have demonstrated that histone demethylases of the KDM3 family are overexpressed in colorectal cancer stem cells, contributing to tumorigenesis via activation of Wnt/β-catenin signaling. IOX1, a known KDM3 inhibitor, disrupts this pathway by targeting the enzymatic activity of KDM3 proteins. Building on this mechanism, researchers have developed IOX1-based PROTACs with optimized linker lengths and enhanced selectivity for KDM3 isoforms. Two lead PROTAC candidates exhibited markedly improved potency, one of which demonstrated up to a 35-fold increase in activity *in vitro* and a 10-fold increase *in vivo*. These findings highlight the therapeutic potential of selective KDM3 degradation as an effective strategy for eradicating colorectal CCSCs and suppressing Wnt-driven tumorigenesis [117].

Nevertheless, several challenges must be addressed to facilitate broader clinical translation of PROTACs, including poor oral bioavailability, potential off-tumor degradation, and limited tissue penetration. The development of next-generation cell-permeable and tissue-specific PROTACs, as well as covalent or light-controllable degraders, is under active investigation [118].

In summary, PROTAC technology is rapidly evolving and holds great promise for CRC treatment, particularly for difficult-to-drug targets. Ongoing research and clinical trials will determine its role in the future therapeutic arsenal against colorectal cancer.

### 4.6 Personalized Medicine and Molecular Profiling

The advent of personalized medicine has seen remarkable advancements, particularly in the ability to tailor treatment based on the unique molecular and genetic profile of each patient’s tumor. The increasing understanding of the biological heterogeneity of colon cancer allows clinicians to identify patient subpopulations likely to benefit from specific targeted therapies or immunotherapies. This process relies on comprehensive genomic screening, most notably through next-generation sequencing (NGS), to detect actionable alterations in genes such as *KRAS*, *NRAS*, *BRAF*, *HER2* amplification, MSI/MMR status, or rare gene fusions. As a result, genomic analysis has become an essential component of CRC clinical decision-making, supporting therapy selection and prognosis estimation.

The development of personalized therapy is based on the identification of molecular biomarkers that have prognostic or predictive (predicting the benefit of a given therapy) significance. In CRC, especially in the advanced stage, the MSI/MMR status is routinely determined; approx. 15% of CRC cases are characterized by MSI-H caused by a deficiency of the mismatch repair system (dMMR), and by gene mutations such as *RAS* (*KRAS*/*NRAS*), *BRAF*^V600E^, or *HER2* gene amplification. Technological advancements enable increasingly comprehensive molecular tumor profiling. It is recommended that in patients with mCRC, the analysis of broader gene panels using next-generation sequencing should be considered to identify rare yet potentially actionable genomic alterations such as fusions or rearrangements of the *neurotrophic tyrosine receptor kinase* (*NTRK*) gene. Their confirmation allows for the use of highly effective tropomyosin receptor kinase (TRK) inhibitors based on agnostic therapy (independent of tumor location) [119–121]. Pharmacogenetic markers, such as mutations in the *dihydropyrimidine dehydrogenase* (*DPYD*) gene, are also being increasingly investigated to identify individuals at elevated risk of severe toxicity from fluoropyrimidine-based chemotherapies such as 5-fluorouracil or capecitabine and to modify therapeutic doses accordingly. Such tests are routinely performed in France and recommended by the European Medicines Agency (EMA). In turn, the presence of the UGT1A1*28/*28 variant for the gene encoding the UDP-glucuronosyltransferase enzyme increases the risk of serious side effects from the hematopoietic system for patients taking irinotecan. Although pharmacogenetic tests are not directly related to the biological characteristics of the tumor, they are part of treatment personalization [119,122].

The use of targeted therapy is also through molecular profiling and individualized treatment planning. In colorectal cancer, the use of the appropriate monoclonal antibody depends on the tumor’s molecular profile. Thus, the effectiveness of anti-EGFR antibodies is not limited only to patients with the wild type of the *RAS* gene but also depends on the tumor’s location. Left-sided (distal) tumors respond better to anti-EGFR antibodies than right-sided ones. Hence, the guidelines suggest preferential use of these drugs, especially in patients with left-sided tumors, while in patients with right-sided colon cancers, alternative options are chosen more often (e.g., anti-VEGF antibodies in the first line of treatment) [123]. Screening for *BRAF* mutations allows for appropriate therapy selection and may significantly improve treatment efficacy. Patients with *BRAF*^*V600E*^ mutation respond differently to targeted therapy compared to those without this mutation [123]. A breakthrough achievement in recent years is the possibility of effective treatment of patients with the *BRAF*^*V600E*^ mutation. Treatment of this group of patients is based on the use of a combination of drugs, such as a BRAF inhibitor (encorafenib) and an anti-EGFR monoclonal antibody (cetuximab). The BEACON study confirmed the efficacy of this personalized approach in treating *BRAF*^*V600E*^-mutant mCRC. Combined treatment with encorafenib and cetuximab, either alone or in combination with binimetinib, significantly improved overall survival and objective response rate compared to standard therapy (irinotecan/FOLFIRI + cetuximab). The combination regimen achieved a median OS of 9.3 months vs. 5.9 months in the control group, confirming improved prognosis for patients with the *BRAF*^*V600E*^ mutation. Additionally, the confirmed ORR was 26.8% in the triple therapy group, 19.5% in the double therapy group, and only 1.8% in the control group [64]. Also, for a small subpopulation of patients with colon cancer who overexpress *HER2* but do not have mutations in the *RAS* and *BRAF* genes, the FDA in 2023 approved the use of a combination therapy consisting of the HER2 kinase inhibitor tucatinib (Tukysa) and the anti-HER2 antibody trastuzumab (Herceptin). Results from clinical trials showed an ORR of 38.1% in patients receiving both tucatinib and trastuzumab; 4% achieved a complete response, and 35% achieved a partial response. Moreover, the median PFS was 8.2 months, and the median OS was 24.1 months [70]. For a small subpopulation of patients with the rare *KRAS*^*G12C*^ mutation, which occurs in only 3%–5% of patients with CRC (but for those with *RASwt* tumours, the incidence is approximately 10%), an alternative targeted therapy has also emerged using a combination of the inhibitor sotorasib with the anti-EGFR antibody panitumumab. This combination has demonstrated efficacy in prolonging PFS and OS compared to standard therapy. The study demonstrated that progression-PFS in the cohort receiving 960 mg of sotorasib plus panitumumab was 5.6 months, compared to 3.9 months in the 240 mg sotorasib group and 2.2 months in the standard of care group (SOC). This indicates that PFS in the combination therapy group more than doubled relative to SOC. Additionally, the ORR in the sotorasib plus panitumumab group was 26.4%, nearly fivefold higher than that observed in the 240 mg sotorasib group (5.7%), while the ORR in the SOC group was 0%. Furthermore, the NCI-MATCH (Molecular Analysis for Therapy Choice) trial highlighted the relevance of precision oncology, showing that biomarker-matched therapies significantly prolonged PFS in patients with metastatic CRC (*p* = 0.01). Despite modest overall efficacy, matched therapeutic approaches, particularly in combination, demonstrated clinical benefit and support the integration of molecular profiling into CRC treatment strategies [71]. Based on the results of the CodeBreak 300 clinical trials (ClinicalTrials.gov identifier NCT05198934), in January 2025, the FDA approved this combination for patients with the *KRAS*^*G12C*^ mutation [124].

Progress in cancer genomics enables precision medicine in CRC treatment. Analysis of mutations in the *KRAS*, *NRAS*, *BRAF* genes, *EGFR* expression level, MSI, and dMMR status enables the selection of appropriate therapy. Next-generation sequencing allows the identification of patients who may benefit from targeted therapies or immunotherapy. The introduction of so-called molecular patient profiles opens the way to individual therapies, increasing the effectiveness of treatment while reducing toxicity [22,23].

## 5 Conclusions: Epidemiological Trends and Therapeutic Directions in Colorectal Cancer

Despite advances in therapy, prevention, and health education, epidemiological data concerning colorectal cancer remain unsatisfactory. Many regions worldwide are experiencing an increase in both the number of new CRC cases and CRC-related deaths, underscoring the growing need for the development of effective treatment strategies and appropriate healthcare resource planning.

In 2020, the number of new CRC cases reached approximately 1.93 million, with nearly 900,000 deaths [125]. By 2021, incidence had increased to 2.19 million cases and 1.044 million deaths [126]. Projections indicate that by 2040, the number of cases will rise by approximately 63%, surpassing 3.15 million annually. The highest relative increases are expected in resource-limited regions such as Sub-Saharan Africa (+95%) and the Eastern Mediterranean Region (+92%) [125]. In the United States, it was estimated that 152,810 new CRC cases would be diagnosed in 2024, with approximately 53,000 associated deaths [127]. Particularly concerning are trends observed in individuals under the age of 55, where both incidence and mortality rates are increasing annually by 1%–2% [128,129]. Notably, CRC has become the leading cause of cancer-related death among men under 50 years of age and the second leading cause among women in the same age group [130]. In Europe, although the overall trend indicates a decline in CRC mortality, 4.8% in men and 9.5% in women since 2018, likely due to the implementation of effective screening programs and advancements in treatment, an opposite tendency is observed in populations under 50 years of age. These increases are particularly evident in countries such as Poland, the United Kingdom, and Italy [131]. At the same time, socio-economic and systemic disparities remain a significant factor directly affecting CRC treatment outcomes. In developing countries with a low Human Development Index (L-HDI), particularly in regions such as Sub-Saharan Africa and South Asia, limited access to colonoscopy, oncological treatment, and healthcare infrastructure results in delayed diagnosis and poorer clinical outcomes [64,132]. The lack of nationwide screening programs, coupled with inadequate funding of public health systems, contributes to persistent disparities in patient survival.

These data clearly indicate that colorectal cancer represents an escalating global health concern. The particularly dynamic increase in CRC incidence in developing countries, coupled with limited access to diagnostic and therapeutic resources, highlights the urgent need to intensify preventive efforts and to develop modern treatment strategies that consider both systemic accessibility and cost-effectiveness.

In parallel, current CRC therapies continue to face several critical limitations, including resistance, toxicity, and lack of efficacy in some patients. The development of personalized medicine, nanotechnology-based delivery systems, and novel organometallic compounds holds promise for transforming the therapeutic landscape. An interdisciplinary approach, integrating pharmacological innovation, genetic profiling, and technological advancements, is essential to improving treatment outcomes and ensuring that emerging therapies are accessible and effective across diverse populations. Therefore, ongoing preclinical and clinical studies are pivotal for addressing existing therapeutic gaps and for developing strategies that are responsive to the evolving epidemiological and socio-economic realities of CRC worldwide.

## Data Availability

Not applicable.

## Abbreviation

APC: Adenomatous Polyposis Coli
AUC: Area Under the Curve
BICR: Blinded Independent Central Review
BRAF: B-Raf Proto-Oncogene, Serine/Threonine Kinase
CRC: Colorectal Cancer
CTLA-4: Cytotoxic T-Lymphocyte Associated Protein 4
dMMR: Deficient Mismatch Repair
DPYD: Dihydropyrimidine Dehydrogenase
EGF: Epidermal Growth Factor
EGFR: Epidermal Growth Factor Receptor
EMA: European Medicines Agency
EPR: Enhanced Permeability and Retention
FDA: Food and Drug Administration
FOXFIRI: Fluorouracil, Leucovorin, Irinotecan
FOLFOX: Fluorouracil, Leucovorin, Oxaliplatin
HER2: Human Epidermal Growth Factor Receptor 2
HIF-1α: Hypoxia-Inducible Factor 1-Alpha
ICIs: Immune Checkpoint Inhibitors
JNK: c-Jun N-terminal Kinases
KRAS: Kirsten Rat Sarcoma Viral Oncogene Homolog
L-HDI: Low Human Development Index
MAPK: Mitogen-Activated Protein Kinase
MMR: Mismatch Repair
MSI-H: Microsatellite Instability High
MSS: Microsatellite Stable
mCRC: Metastatic Colorectal Cancer
NGS: Next-Generation Sequencing
NRAS: Neuroblastoma RAS Viral Oncogene Homolog
NTRK: Neurotrophic Tyrosine Receptor Kinase
OS: Overall Survival
ORR: Objective Response Rate
PD-1: Programmed Cell Death Protein 1
PFS: Progression-Free Survival
RAS: Rat Sarcoma
ROS: Reactive Oxygen Species
SOC: Standard of Care
TGF-β: Transforming Growth Factor Beta
TNM: Tumor Node Metastasis
TP53: Tumor Protein P53
TRK: Tropomyosin Receptor Kinase
TrxR: Thioredoxin Reductase
UGT1A1: UDP-Glucuronosyltransferase 1A1
VEGF: Vascular Endothelial Growth Factor
VEGFR: Vascular Endothelial Growth Factor Receptor
WHO: World Health Organization
XELOX: Capecitabine + Oxaliplatin
